# The Effect of Oral Supplementation of Vitamin D_3_ on Serum Levels of Vitamin D: A Review

**DOI:** 10.4172/2161-1165.1000148

**Published:** 2014-02-12

**Authors:** Francesca Jarrett, Gloria Michelle Ducasa, David B Buller, Marianne Berwick

**Affiliations:** 1 Special Student, University of New Mexico, Albuquerque, New Mexico, USA; 2 PREP Program, University of New Mexico, Albuquerque, New Mexico, USA; 3 Klein Buendel, Golden, Colorado, USA; 4 Professor, Department of Internal Medicine and Dermatology, University of New Mexico, Albuquerque, New Mexico, USA

**Keywords:** Ultraviolet radiation, Cholecalciferol, Serum levels

## Abstract

Due to the strong interest in the role of vitamin D in health, we tried to find data to illustrate the relationship between oral supplementation and change in serum levels of vitamin D. We reviewed the literature of randomized, placebo-controlled trials of oral supplementation and serum levels of vitamin D through 2014. We found 25 informative studies which showed a significant dose-response between oral supplementation and serum levels of vitamin D with an r^2^ of 0.61.These data are consistent with single studies and meta-analyses.In conclusion, the data show a consistent relationship that appears to be independent of multiple confounders.

## Introduction

Recently there has been a resurgence of interest in the role of vitamin D_3_, or cholecalciferol, supplementation in disease prevention and health maintenance. Systematic reviews have recently been published with a focus on specific groups, such as those over 50 [1] or by body mass index [2]. Studies have been carried out on serum vitamin D levels to evaluate the role of supplementation on risk for multiple health conditions, ranging from bone health to cancer.

However, the benefits and risks of cholecalciferol supplementation are under debate after the publication of a 2010 Institute of Medicine Report that recommended supplementary and dietary reference levels for Vitamin D [3]. From an epidemiological perspective, cholecalciferol is unique, as it can be obtained not only through food and supplementation, but also from ultraviolet radiation, specifically UVB (280-320 nm) [4]. Thus, some groups have suggested that people increase time in the sun and forgo sunscreen in an effort to increase their serum 25-(OH)D levels [5]. However, because over exposure to UVB rays can also lead to an increase in skin cancer incidence, the health effects associated with UV exposure may be problematic.

Oral supplementation of Vitamin D_3_ may be a safe alternative to UVB and should be easy to regulate to achieve optimum dosage [6]. Unfortunately, outside of single studies, there is little research that evaluates the dose-response relationship between oral intake of cholecalciferol and subsequent serum levels of vitamin D.Because of this knowledge gap, we conducted a literature review to gain perspective on current knowledge about the dose-response of serum vitamin D with supplementation.

## Methods

**Figure 1:**
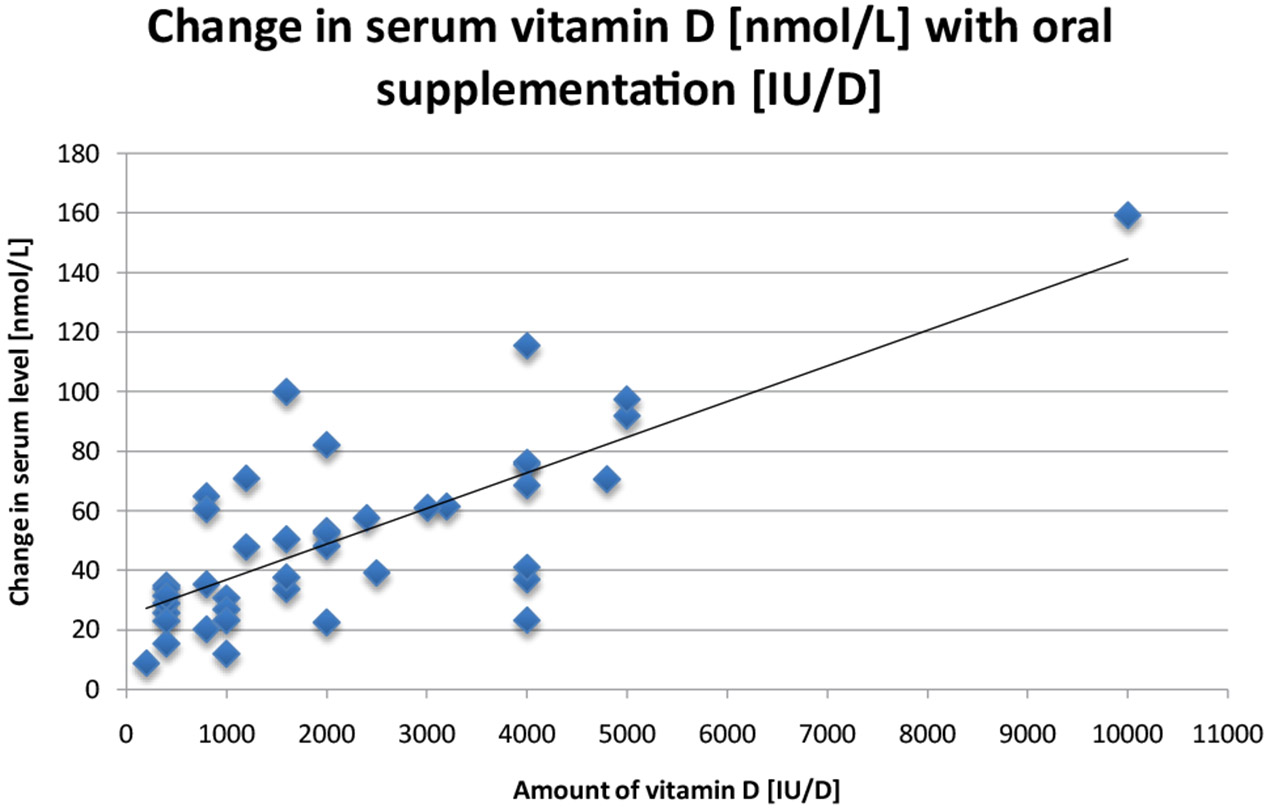
Change in serum vitamin D [nmol/L] with oral supplementation [IU/D] in 25 studies.

We reviewed studies identified through a combination of personal archives and the National Library of Medicine database PubMed using the search terms “vitamin D supplementation,” “vitamin D serum” “randomized trials of vitamin D” and “25-(OH)D.” The earliest eligible study available was from 1991 and the most recent was from 2013. Studies were excluded for incomplete, or inaccessible data, leaving 36 studies that were included in this review. Of these 36 studies, 25 were analyzed (Table 1) and evaluated through graphical analysis (Figure 1), and 11 were listed separately (Table 2) because of irregular dose patterns, such as a one-time bolus intervention.

The studies we reviewed were chosen from available literature addressing vitamin D and met pre-determined criteria for involvement.These criteria included information as to the dose of oral supplementation of cholecalciferol (vitamin D_3_) and serum levels of 25-(OH)D at baseline and post-intervention and included placebo groups for comparison (Tables 1 and 2). We found that many studies looked at the effect of vitamin D supplementation and dietary intake on disease outcome. Sixteen studies meeting our criteria evaluated healthy individuals [7-22]. Nine studies meeting our criteria evaluated individuals with disease included individuals with vitamin D deficiency [23,24], individuals with diabetes [25-27], relapsing multiple sclerosis patients [28], individuals with early stage prostate cancer [29], patients with previous hip fractures [30], and Parkinson Disease [31]. The number of studies that included the necessary information on serum levels pre- and post-supplementation were limited, and only a few addressed serum vitamin D levels exclusively in response to oral supplementation.

Although we wished to perform a meta-analysis, we found that the data were too heterogeneous for valid statistical evaluation. Some of the factors that contributed to the heterogeneity included varying geographical latitude, age, pre-existing medical conditions, dose size, and calcium supplementation, frequency of dose, follow-up time, and overall quality of the study. In addition, we found that several studies did not include crucial information, such as standard deviation or baseline levels, because vitamin D was not the primary focus of their research. Studies that were not placebo-controlled, randomized, or had an inadequate sample size (i.e., fewer than 20) were eliminated from our main analysis. It should be noted that seven of the studies gave weekly doses of cholecalciferol (Table 2) while three others administered one-time only bolus doses. These two groups were evaluated separately and not included in the daily dose results.

In order to determine the change in serum vitamin D per amount of oral supplementation, we subtracted the baseline serum level from the post-intervention serum level and correlated that with the amount of oral vitamin D given. This information was calculated by linear regression using change from baseline serum vitamin D and with duration included as both a quadratic and linear term using SAS 9.3 (Carr, NC).

## Results

Analyses of the best available data show a clear trend. Figure 1, which shows the data from the twenty most reliable and homogeneous studies [7-26], exhibits a positive correlation between amounts of oral vitamin D administered and change in serum vitamin D levels. Figure 1 shows the change in vitamin D serum levels from the most rigorous studies that gave daily doses of cholecalciferol. These values are highly significantly correlated with a *P*-value<0.001 and an r^2^ of 0.61. We did not analyze the data from Table 2 statistically because the data were too few for meaningful analysis.

## Discussion

These results demonstrate the overall coherence of the more generalizable studies, all of which were double blind and had an adequate sample size. Interestingly, our simple model is quite robust. There is a relatively strong dose-response between the amount of supplement and the change in serum vitamin D status.Surprisingly, results did not change significantly when we restricted them to studies of healthy individuals or to studies with intervention of 6 months or more. From the large scatter noted in Figure 1, it is clear that a great deal more information needs to be evaluated regarding the appropriate dose if vitamin D_3_ to raise serum vitamin D to a specific level. In addition, there is a clear need for additional placebo-controlled, double-blind studies to be conducted on the effects of serum 25-(OH)D levels in response to oral supplementation. One of the main limitations to our research was the fact that there are actually few randomized studies of cholecalciferol supplementation. To be useful for study of the effect of cholecalciferol on health and disease outcomes as well as clinical utility, it is important to know how different doses of vitamin D change serum vitamin D. Thus more studies need to be conducted on the oral supplementation of cholecalciferol before the scientific community can understand the importance of vitamin D supplementation to health.

Limitations include the heterogeneous nature of the studies and the fact that our analysis did not take into account the climate zone in which a person lives, or the role of age, body mass index, activity and diet in determining serum levels of vitamin D. In addition, the linear regression and slope determined using Figure 1, do not account for the “plateau” of serum vitamin D levels at high concentrations (e.g., 23). However, as a beginning, our study provides a baseline from which develop a more precise evaluation. This is especially important in light of the aging US population, the recent increase in interest in the role of vitamin D in disease prevention and the urgent need to carefully understand the role of vitamin D in health-whether it is a symptom of poor health or a means to better health.

## Figures and Tables

**Table 1:** Randomized studies of daily vitamin D [IU/D] with change in serum level [nmol/L] over baseline.

Study Number and Author	Location	Subjects [number, age, and sex]	Length of Study	Amount of Supplemental Vitamin D Given	Serum levels-Baseline and After [nmol/L]	Change in serum level [nmol/L]
Biancuzzo RM et al. [7]	Boston, Massachusetts	34 healthy adults, 18-79	11 weeks	1000 IU/D	Baseline: 53.2 After: 83.9	30.7
Al-Shaar L et al. [8]	Lebanon	336 healthy adolescents	1 year	200 IU/D 2000 IU/D	200 Baseline: 37.4 200 After: 46.4 2000 Baseline: 37.4 2000 After: 90.6	200: 8.9 2000: 53.2
Ng K et al. [9]	Boston, Massachusetts	328 African American, 30-80 years old	3 months	1000 IU/D 2000 IU/D 4000 IU/D	1000 Baseline: 40.4 1000 After: 74.1 2000 Baseline: 34.7 2000 After: 86.9 4000 Baseline: 39.2 4000 After: 114.6	1000: 26.9 2000: 48.2 4000: 75.6
Ala-Houhala MJ et al. [10]	Tampere, Finland	33 healthy hospital employees	4 weeks	800 IU/D	Baseline: 53.5 After: 73.7	20.2
Holmlund-Suila E et al. [11]	Helsinki, Finland	113 healthy newborn infants from a single hospital	10 Weeks	400, 1200. Or 1600 IU/D	400 Baseline: 53 400 After: 88 1200 Baseline: 53 1200 After: 124 1600 Baseline: 53 1600 After: 153	400: 35 1200: 71 1600: 100
Gepner AD et al. [12]	Madison, Wisconsin	114 healthy post-menopausal women mean age 63.9,	4 Months	2500 IU/D	Baseline: 78.1 After: 117.3	39.2
Holvik K et al. [13]	Oslo, Norway	55 subjects. Healthy adults], average age 28 years, 63.6% women]	4 weeks	400 IU/D	Baseline: 44.3 After: 78.4	34.1
Toss G et al. [14]	Linköping, Sweden	45 subjects of which 32 were female, aged 55-84	1 Year	1600 IU/D	Baseline: 50.4 After: 84.2	33.8
Dong Y et al. [15]	Augusta, Georgia	49 Normotensive African American boys and girls, mean age 16.3	16 Weeks	400 IU/D or 2000 IU/D	400 Baseline: 34 400 After: 59.8 2000 Baseline: 33.1 2000 After: 85.7	400: 25.8 2000: 52.6
Pfeifer M et al. [16]	Bad Prymont, Germany Graz, Austria	114 healthy men and women, 70+ years	1 year	400 IU/D	Baseline: 55 After: 84	29
Holick M F et al. [17]	Boston, Massachusetts	68 healthy, different racial/ethnic groups, 18-84 years old	11 weeks	1000 IU/D	Baseline: 48.9 After: 72.1	23.2
Heany R P et al. [18]	Omaha, Nebraska	67 healthy men, average age 38.7	20 weeks	1,000, 5,000 or 10,000 IU/D	1000 baseline: 72.1 1000 after: 84.1 5,000 Baseline: 69.3 5,00 After: 161.2 10,000 Baseline: 65.6 10,000 After: 225.0	1000: 12 5,000: 91.9 10,000: 159.4
Trang H M et al. [19]	Toronto, Canada	72 subjects, mean age 38	14 days	4000 IU/D	Baseline: 41.3 After: 64.6	23.3
Lips P et al. [20]	Amsterdam, Netherlands	2578 people, including 1916 women and 662 men, mean age 80, with no major health problems	3.5 years	4000 IU/D	Baseline: 23 After: 60	37
Chapuy MC et al. [21]	Lyon, France	142 Healthy, ambulatory women aged 84 ± 6 years	18 months	800 IU/D	Baseline: 39.9 After: 104.8	64.9
Dawson-Hughes B et al. [22]	Massachusetts	333 healthy, postmenopausal women, mean age 61.4	1 year	400 IU/D	Summer baseline: 81.6 Post summer: 97 Winter baseline: 60.6 Post winter: 92.1	Summer: 15.4 Winter: 31.5
Gallagher JC et al. [23]	Omaha, Nebraska	163 postmenopausal white females with vitamin D insufficiency, mean age 67, divided into 8 study groups of 20 or 21 participants	1 year	400, 800, 1600, 2400, 3200, 4000, or 4800 IU/D	400 Baseline: 37.8 400 After: 60.9 800 Baseline: 39.0 800 After: 74.4 1600 Baseline: 37.4 1600 After: 87.9 2400 Baseline: 38.2 2400 After: 95.8 3200 Baseline: 39.8 3200 After: 101.3 4000 Baseline: 37.2 4000 After: 105.8 4800 Baseline: 38.6 4800 After: 109.3	400: 23.1 800: 35.4 1600: 50.5 2400: 57.6 3200: 61.5 4000: 68.6 4800: 70.7
Bogh MK et al. [24]	Malmö, Sweden	32 vitamin D deficient patients from a primary care center in Malmö, Sweden, mean age of 32	6 weeks	1600 IU/D	Baseline: 23.3 After: 60.6	37.7
Al-Daghri NM et al. [25]	Riyadh, Kingdom of Saudi Arabia	92 total, 58 women, median age 56.6 and 34 men, medial age 51.2. All had Diabetes Mellitus Type 2	18 Months	2000 IU/D	Baseline: 32.2 After: 54.7	22.5
Harris SS et al. [26]	Boston, Massachusetts	89, Overweight or obese African Americans with prediabetes or diabetes	12 Weeks	4000 IU/D	Baseline: 40 After: 81	41
Yiu Y et al. [27]	Hong Kong, China	100 type 2 DM patients with 25 [OH]D concentration <30 ng.mL	12 weeks	5000 IU/D	Baseline: 54.9 After: 152.4	97.5
Pierrot-Deseilligny C et al. [28]	Paris, France	156 Relapsing-remitting multiple sclerosis patients under first line immunomodulatory therapy and initial serum levels less than 100 nmol/L	29.1 Months	3010 IU/D	Baseline: 49 After: 110	61
Garrett-Mayer E et al. [29]	Columbia, South Carolina	47 patients, 12 African American [mean age 63.2] and 35 White men [mean age 65.3] with early-stage low-risk prostate cancer	1 year	4000 IU/D	Caucasian Baseline: 91.6 Caucasian After: 167.9 African American Baseline: 53.4 African American After: 168.9	Caucasian: 76.3 African American: 115.5
Bischoff-Ferrari HA et al. [30]	Zurich, Switzerland	173 patients with previous hip fracture age >65 [average age 84 years] 79% Women	1 year	800 IU/D or 2000 IU/D	800 Baseline: 31.5 800 After: 92.0 2000 Baseline: 34.1 2000 After: 116.21	800: 60.6 2000: 82.2
Suzuki M et al. [31]	Tokyo, Japan	114 patients with Parkinson’s Disease, aged 45–85	1 year	1200 IU/D	Baseline: 56.2 After: 104.1	47.9

**Table 2:** Randomized studies of vitamin D [IU] that gave weekly, monthly or bolus supplementation [nmol/L].

Study Number and Author	Location of Study	Subjects [number, age, and sex]	Length of Study	Amount of Supplemental Vitamin D Given	Serum levels-Baseline and After [nmol/L]	Change in serum level [nmol/L]
Matias PJet al. [32]	Portugal	158 Hemodialysis Patients	6 months	50,000 IU/week [25[OH]D<15 ng/mL] 10,000 IU/week [25[OH] 16-30 ng/mL] 2,700 IU/week [25[OH]D >30ng/mL	Baseline: 55.7 After: 104.8	49.1
Goswami Ret al. [33]	New Delhi, India	173 Healthy Asian Indian females with a mean age of 21.7	6 Months	60,000 IU/ week	Baseline: 57.9 After: 186.2	128.3
Alvarez JAet al. [34]	Atlanta, GA	46 patients with stage 2 & 3 CKD 18-90 years	12 months	50,000 IU/week for 12 weeks 50,000 IU/every other week for 40 weeks	Baseline: 67.4 After: 116.6	49.2
Markmann Pet al. [35]	Odense, Denmark	52 Chronic Kidney Disease patients, male and female, mean age 71	8 Weeks	40,000 IU/ week	Baseline: 23.8 After: 141.6	117.8
Armas LAet al. [36]	Omaha, Nebraska	Patients with Stage 5 Chronic Kidney Disease	15 Weeks	10,333 IU/week	Baseline: 33.2 After: 92.1	58.9
Lips Pet al. [37]	Subjects came from Mexico Washington, Indiana, Nebraska, Netherlands, Germany, and, Canada	226 men and women, mean age 78.5, who were vitamin D deficient	16 Weeks	8400 IU/week	Baseline: 34.7 After: 65.4	30.7
Tokmak Fet al. [38]	North Rhine-Westphalia, Germany	64 haemodialysis patients	9 months	20,000 IU/week	Baseline: 16.7 After: 79.5	62.8
Jakopin Eet al. [39]	Slovenia	101 Hemodialysis patients [52 men, 49 women] Average age 63.3 years	24 months	40,000 IU/month	Baseline: 28.6 After: 54.9	26.3
Witham MCet al. [40]	Dundee, United Kingdom	74 subjects with a history of myocardial infarction, average age 66 years	6 months	100,000 IU Bolus every two months	Baseline: 49 After: 62	13
Grossman REet al. [41]	Atlanta, Georgia	30 Adults in the hospital for Cystic Fibrosis Pulmonary Exacerbation, mean age 24.9, both male and female, all Caucasian	12 Weeks	One time 250,000 IU Bolus	Baseline: 76.4 After: 91.6	15.2
Tran Bet al. [42]	Australia	615 population-based, 60-84 years old	1 year	30,000 IU/month 60,000 IU/month	30,000 Baseline: 41.6 30,000 After: 63.9 60,000 Baseline: 41.7 60,000 After: 77.9	30,000: 22.3 60,000: 36.1
